# Display of Porcine Epidemic Diarrhea Virus Spike Protein on Baculovirus to Improve Immunogenicity and Protective Efficacy

**DOI:** 10.3390/v10070346

**Published:** 2018-06-27

**Authors:** Chia-Yu Chang, Wei-Ting Hsu, Yu-Chan Chao, Hui-Wen Chang

**Affiliations:** 1 School of Veterinary Medicine, National Taiwan University, Taipei 106, Taiwan; f04644007@ntu.edu.tw; 2 Institute of Molecular Biology, Academia Sinica, Nankang, Taipei 115, Taiwan; waitinglovekeroro@gmail.com; 3 Graduate Institute of Molecular and Comparative Pathobiology, School of Veterinary Medicine, National Taiwan University, Taipei 106, Taiwan

**Keywords:** baculovirus display system, PEDV, spike vaccine, intramuscular injection

## Abstract

A new variant of the porcine epidemic diarrhea virus (PEDV) is an emerging swine disease, killing considerable numbers of neonatal piglets in North America and Asia in recent years. To generate immunogens mimicking the complex spike (S) protein folding with proper posttranslational modification to mount a robust immune response against the highly virulent PEDV, two baculoviruses displaying the full-length S protein (S-Bac) and the S1 protein (S1-Bac) of the virulent Taiwan genotype 2b (G2b) PEDV Pintung 52 (PEDV-PT) strain were constructed. Intramuscular immunizations of mice and piglets with the S-Bac and S1-Bac demonstrated significantly higher levels of systemic anti-PEDV S-specific IgG, as compared with control group. Our results also showed that piglets in the S-Bac group elicited superior PEDV-specific neutralizing antibodies than those of the S1-Bac and control groups. The highly virulent PEDV-PT strain challenge experiment showed that piglets immunized with S-Bac and S1-Bac showed milder clinical symptoms with significantly less fecal viral shedding as compared with non-immunized control piglets. More importantly, piglets immunized with the S-Bac exhibited no to mild clinical signs, with a delayed, minimal viral shedding. Our results demonstrated that the S-Bac could serve as a safe, easy to manipulate, and effective vaccine candidate against the PEDV infection.

## 1. Introduction

Porcine epidemic diarrhea (PED) is a highly contagious swine disease characterized by acute watery diarrhea and vomiting in piglets [1]. The PED was first identified in the 1970s in Europe and subsequently became an endemic disease with sporadic outbreaks in Asia and Europe [2,3]. Since late 2010, the highly virulent porcine epidemic diarrhea virus (PEDV) has emerged and has attacked neonatal piglets in China [4]. In 2013, outbreaks of highly virulent PED were reported in North America and East Asia [5,6], including in Taiwan [7], resulting in dramatic economic losses in swine industries. A lot of effort has been made to develop vaccines for controlling the epidemic of PED [6,8,9,10]. However, no effective commercialized vaccine is available for controlling PED worldwide. A valid, safe, and cost-effective vaccine for controlling PEDVs is therefore urgently needed. Additionally, the phylogenic analysis of new Taiwan PEDV strains showed high sequence identities with other G2b PEDVs in other countries [7]. Therefore, development of the Taiwan G2b PEDV-based vaccines would be very helpful for controlling and reducing the disease burden.

Porcine epidemic diarrhea virus belongs to family Coronaviridae, subfamily Coronavirinae and genus Alphacoronavirus [1]. The virus has a single, 28 kb, positive strain RNA genome encoding seven open reading frames (ORF): the ORF1a and ORF1b genes encode the replicase; the ORF3 gene encodes the accessory protein; the ORF2, ORF4, ORF5, and ORF6 genes encode four structural proteins, namely spike (S), envelope (E), nucleocapsid (N), and membrane (M) protein [11]. Among the structure proteins, the S protein, which is responsible for virus-host recognition, virus internalization, and neutralizing antibody induction, is the principle antigenic determinant [1]. The naturally assembled S protein forms a homotrimeric structure with several predicted glycosylation sites [12]. This glycoprotein can further be divided into S1 and S2 domains by specific cleavage site [13]. The S1 domain contributes to receptor recognition, enteropathogenicity, and immunogenicity, as it contains the dominant neutralizing epitopes, thus being the main target for vaccine development [14,15]. Linking with the S1, the S2 domain anchors on the viral membrane and triggers membrane fusion [16]. Several neutralizing epitopes on the S protein of PEDV have been described, including the CO-26K-equivalent epitope (COE epitope) [15], the antigen epitope motif recognized by monoclonal antibody 2C10 at C terminal end of S protein [17,18], and the S1D domain [19]. Therefore, S protein is considered a major target for PEDV vaccine development [9,20].

The baculovirus expressing system has long been utilized for different biological purposes, for instance, recombinant protein production [21], vaccine manufacturing, and gene therapy [22,23]. Among various strains, Autographa californica multiple nucleopolyhedrovirus (AcMNPV) is the most popular strain for laboratory applications. The high protein productivity, large capacity and flexibility to accommodate long insertions [24], high biosafety for vertebrates, and delicate post-translation modifications are the advantages of the baculovirus expressing system [25]. Furthermore, as a vaccine candidate, the viral particle of baculovirus is able to evoke robust innate immune responses by regulating cytokines [26], as well as serving an adjuvant role in promoting B cell and T cell activation to improve the humoral immune responses [27]. Baculovirus is a very stable DNA virus, which can be stored at 4 °C for up to 4 years without significant change in virus titers [28]. Also, this virus is easy to propagate, with high titers around 10^8^–10^9^ TCID50/mL. Recently, baculovirus-expressing viral surface proteins has become a novel strategy for recreating complex protein folding and functional properties [29,30]. Given that baculovirus is able to mount a robust immune response [26,31], the baculovirus displaying a heterologous protein on its surface could be a novel tool for the development of new generation of vaccines. In this context, several immunogens have been generated using the baculovirus display strategy and proven to be able to induce strong humoral immunity and protection against infectious diseases [32,33,34].

In the present study, taking the advantages of the recombinant baculovirus display system, the PEDV full-length S protein and PEDV S1 protein named S-Bac and S1-Bac, were constructed and displayed on baculovirus. The immunogenicities of S-Bac and S1-Bac were compared and evaluated in mice and pigs by intramuscular (IM) injections without cooperating with adjuvants. The protective efficacy that these baculovirus display vaccines against the highly virulent PEDV-PT strain were also evaluated.

## 2. Materials and Methods

### 2.1. Viruses and Cell Lines

The highly virulent PEDV Pintung 52 passage 5 (PEDV-PT-P5) (GenBank Accession No. KY929405) viral stock was used for preparation of the PEDV-PT passage 6 (PEDV-PT-P6) and PEDV-PT passage 7 (PEDV-PT-P7) in Vero cells (American Type Culture Collection (ATCC) No. CRL-1586) as previously described [35]. A viral challenge stock of PEDV-PT-P6&7 was prepared by admixing 1:1 ratio of the PEDV-PT-P6 and PEDV-PT-P7 supernatants. The titer of the PEDV-PT-P6&7 viral stock was determined as 10^5^ TCID50/mL by performing a 10-fold serial diluted inoculation on Vero cells.

### 2.2. Plasmid Construction 

**Figure 1:**
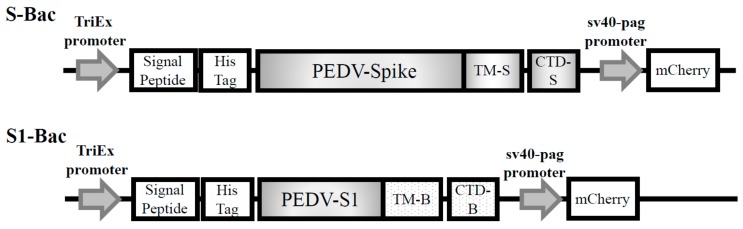
The organization of the expression cassette and the construction maps of the plasmids, pTriEx-S and pTriEx-S1, for generation of membrane-anchored recombinant baculovirus, S-Bac and S1-Bac. The full-length spike (S) and S1 genes are driven by the TriEx promoter, followed by the GP64 signal protein and 6xHis-tag. The pTriEx-S contains the codon optimized full-length S gene derived from the Taiwan G2b PEDV-PT strain with its original PEDV S transmembrane domain (TM-S) and the PEDV S cytoplasmic domain (CTD-S) for membrane anchoring. The pTriEx-S1 has the codon optimized S1 gene associated with GP64 transmembrane domain (TM-B) and the GP64 cytoplasmic domain (CTD-B) for membrane anchoring. Both constructs were also inserted with an mCherry fluorescent protein gene driven by the SV40-pag promoter as a reporter.

The nucleotide sequence of S gene derived from the Taiwan G2b PEDV-PT strain (Genbank accession No. KP276252) was codon optimized for insect cells and synthesized by ProTech (ProTech, Taipei, Taiwan). The PEDV full length spike (S) and S1 genes were cloned into pTriEx-3 plasmid (Novagen, Merck Biosciences, Darmstadt, Germany), bringing pTriEx-S and pTriEx-S1, respectively. The pTriEx-3 plasmid contains tripartite p10, CMV and T7 promoters for the convenient expression in insect, mammalian, and bacterial cells [36,37]. The full-length S and S1 protein sequences were driven by TriEx promoter with 6xHis-tag in plasmids pTriEx-S and pTriEx-S1, respectively (Figure 1). The mCherry gene was driven by the binary sv40-pag promoter for emitting reporter fluorescence in Sf21 and mammalian cells. The plasmids were constructed according to the instruction manual of the In-Fusion^®^ HD Cloning Kit (Clontech Laboratories Inc., Fremont, CA, USA).

### 2.3. Recombinant Baculovirus Preparation

Plasmids pTriEx-S and pTriEx-S1 were co-transfected with FlashBAC™ (Mirus, Madison, WI, USA) DNA into Sf21 cells by Cellfectin (Life Technologies, Carlsbad, CA, USA) to further generate recombinant baculoviruses, S-Bac and S1-Bac. The expression of mCherry gene product and 6xHis-tag are used to trace proper viral infection and protein expression. The S-Bac and S1-Bac virus clones with high titers were selected and used for subsequently recombinant baculovirus production.

### 2.4. Western Blotting

The infected cell lysates were subjected to gradient sodium dodecyl sulfate (SDS)-polyacrylamide electrophoresis (PAGE) gel (HR gradient gel solution, TOOLS, Taipei, Taiwan). After electrophoresis, proteins were transferred to PVDF membranes. The protein signals were detected by using mouse anti-6xHis-tag monoclonal antibody (1:5000 dilution, EnoGene, New York, NY, USA). Then, the goat anti-mouse IgG conjugated to HRP (1:5000 dilution, Invitrogen, Carlsbad, CA, USA) were used as the secondary antibodies for signal detection. The protein bands were detected by using the Clarity™ Western ECL Blotting Substrates (Bio-Rad, Hercules, CA, USA) using Classic Blue Autoradiography film BX (Life Science, Valley Park, MO, USA).

### 2.5. Characterizations of S-Bac and S1-Bac by Electron Microscopy (EM)

Supernatants were collected from the S-Bac-inoculated and S1-Bac-inoculated Sf21 cells. The cell debris was coarsely removed by centrifugation at 10,000 rpm for 30 min, then the supernatants were collected and subjected onto the 25% (*w*/*w*) sucrose cushion in SW28 tubes (Beckman, Brea, CA, USA) for centrifugation at 24,000 rpm for 80 min in 4 °C to obtain the viral pellet. After discarding the supernatant, the viral pellets were resuspended with 1 mL PBS, then subjected to a 25–60% (*w*/*w*) sucrose gradient at 28,000 rpm for 3 h. Viral particles were collected and washed with PBS to remove sucrose. These purified viral particles were then fixed, labeled with anti-His immunogold, and visualized by electron microscopy (EM) with negative staining as described in previous studies [38,39]. Briefly, an aliquot of 10 µL virus particles preparation was loaded onto a carbon-coated grid, letting stand for 5 min. Grids were then stained with 2% phosphotungstic acid (PTA) for 1 min, then the excess PTA was drained and completely dried out, and the grids were examined directly under EM. For immunogold labeling, virus particles were loaded onto a collodion-coated EM grid for 5 min. After the removal of excess viral particles by gently blotting with filter paper at the edge of the grid, an anti-His tag antibody (Invitrogen) was added onto the grid and incubated for 1 h at room temperature. Grids then underwent 10 s wash six times in PBS at room temperature and were incubated with 6 nm gold-conjugated anti-mouse IgG for 1 h. After six washes in PBS, the grids were stained with 2% PTA for 1 min, then drained and dried out, then examined under the EM.

### 2.6. Immunization Program of Mice

Twelve Balb/c mice were randomly divided into three groups: control, S-Bac, and S1-Bac groups. The mice were immunized intramuscularly on the thigh with either S-Bac or S1-Bac, by a dosage of 200 µL of 10^9^ TCID50/mL per shot. The mice in the control group were injected with 200 µL of the cell culture medium of Sf21. The injections were performed two times with 2-week intervals. The blood was collected at day 0 (pre-priming), 14 (2 weeks after priming), and 28 (2 weeks post-boosting) for evaluating the change of PEDV-specific IgG.

### 2.7. Immunization Program of Piglets

Fifteen five-week old, Large White × Duroc, crossbred, PEDV-seronegative, and fecal PEDV shedding negative pigs were screened for our experimental applications. All piglets were labeled by ear tags, stochastically separated into three groups, including the control group, the S1-Bac IM injection group, and the S-Bac IM injection group, and housed in three separate rooms. Each group of pigs was intramuscularly injected with 2 mL control medium, 2 mL of 10^9^ TCID50/mL S1-Bac, or 2 mL of 10^9^ TCID50/mL S-Bac on both sides of the thigh, two times at a two-week interval. At day 28, all pigs were orally challenged with 5 mL of 10^5^ TCID50/mL PEDV-PT-P6&7. After challenging, the clinical signs were scored, and rectal swabs were collected every day to monitor the viral shedding and mucosal IgA. Blood was collected every two weeks in order to evaluate the PEDV S protein-specific plasma IgG. All animal experimental procedures performed on the animals were reviewed and approved by the Institutional Animal Care and Use Committee of National Taiwan University (Taipei, Taiwan, NTU106EL-00054).

### 2.8. Stool Scoring

The clinical signs of each pig were observed and recorded every day. The condition of diarrhea associated with PEDV challenging was scored into four levels: 0, normal stool; 1, loose consistency of the stool; 2, semi-fluid consistency of the stool; 3, watery diarrhea. Additionally, pigs of each group were weighed every two weeks.

### 2.9. ELISA for Detecting Systemic IgG

The purified recombinant S protein expressed by HEK 293 cells as previously described [40] was coated on the ninety-six well, Nunc maxi-soap plate (Thermo Fisher Scientific, Waltham, MA, USA) with the concentration of 2 µg/µL diluted in coating buffer (KPL, SeraCare, Milford, MA, USA) at 4 °C for 16 h. The S-coated plates were firstly washed six times with 200 µL washing buffer (KPL, SeraCare) and blocked with blocking buffer (KPL, SeraCare) at room temperature (RT) for 1 h. After centrifuging at 3000 rpm for 30 min and removing the blood cells, the blood samples of mice and pigs were diluted 40-fold in blocking buffer (KPL, SeraCare) and followed by 1 h incubation on the S-coated plates at RT for single well measurements. The hyper-immunized serum from mouse and pig were utilized as positive control. The plates were washed six times in 200 µL washing buffer (KPL, SeraCare) after incubation, and the antibodies were detected by either using 1000× diluted horseradish peroxidase (HRP)-conjugated goat anti-mouse IgG (KPL, SeraCare) or HRP-conjugated goat anti-pig IgG (KPL, SeraCare) in blocking buffer (KPL, SeraCare). After 1 h incubation, the plates were washed six times with 200 µL washing buffer (KPL, SeraCare). Fifty microliters of ABTS^®^ Peroxidase Substrate System (KPL, SeraCare) was added each well at RT for 10 min. The reaction was stopped by adding 50 µL stopping solution (KPL, SeraCare), and the optical density (OD) was read at a wavelength of 405 nm by EMax Plus Microplate Reader (Molecular Devices, Crawley, UK). The result was expressed as sample to positive ratio (S/P ratio). The S/P ratio represented the difference between the OD values of sample and negative control divided by the difference between OD value of positive and negative controls.

### 2.10. ELISA for Detecting Mucosal IgA

Each rectal swab was resuspended in 1 mL PBS and was two-fold diluted in blocking buffer (KPL, SeraCare). The ELISA procedures were as mentioned above, with the modifications of incubating the suspension supernatants for 16–18 h under 4 °C and followed by a 1 h incubation of secondary antibody of goat anti-pig IgA (KPL, SeraCare) to detect the fecal IgA with 20 min of coloration time. The fecal swab from challenged pig was used as the positive control.

### 2.11. RNA Extraction, cDNA Synthesis, and Probed Quantitative Real-Time PCR

Each rectal swab was resuspended in 1 mL PBS, and 200 µL of each supernatant was used for RNA extraction. The procedures of RNA extraction were performed by QIAcube HT (Qiagen, Chatsworth, CA, USA) using a QIAamp cador Pathogen Mini Kit (Qiagen), according to the manufacturer’s instructions. Complementary DNA (cDNA) synthesis was performed by reverse transcription using the QuantiNova Probe PCR Kit (Qiagen). The real-time PCR was modified according to a previously established method [41] using the specific probe (3′- FAM-TGYYACCAYYACCACGACTCCTGC-BHQ1-5′), PEDV-N forward primer (3′-CGCAAAGACTGAACCCACTAAC-5′), and PEDV-N reverse primer (3′- TTGCCTCTGTTGTTACTTGGAGAT-5′). The real-time PCR condition was 95 °C for 2 min and 45 cycles of 95 °C for 15 s and 55 °C for 15 s.

### 2.12. Neutralizing Assay

The plasma samples of each pig were incubated at 56 °C for 30 min to inactivate the complement. The procedure of the neutralizing assay was performed as previously published with some modifications [35]. Briefly, the plasma samples were diluted from 10-fold to 320-fold in Dulbecco’s modified Eagle’s medium (DMEM) (Gibco, Gaithersburg, MD, USA). Fifty microliters of the diluted plasma samples was mixed with an equal volume of 200 TCID50/mL of PEDV-PT-P6&7. After incubating at 37 °C for 1 h, the mixture was added onto the Vero cells grown on 96-well plates, with a 90% confluence. The cells were incubated at 37 °C for 1 h and followed by two washes of DMEM and replaced by 100 µL of the fresh post-inoculation (PI) medium, which contained DMEM (Gibco, Gaithersburg, MD, USA) supplemented with tryptose phosphate broth (0.3%) (Sigma, St. Louis, MO, USA), yeast extract (0.02%) (Acumedia, Lansing, CA, USA), and 10 μg/mL of trypsin (Gibco, Gaithersburg, MD, USA). The cytopathic effect (CPE) was observed at 24 h. The neutralizing titers of each plasma were calculated as the reciprocal of the highest dilutions without CPE.

### 2.13. Statistical Analysis

The results of IgG level, IgA level, body weight, antibody titer, and viral shedding were statistically compiled with SAS 9.4 (Statistical Analysis System, SAS Institute Inc., Cary, NC, USA). The differences between each group were compared by one-way analysis of variance (ANOVA). The significance was determined to have a *p*-value < 0.05 (*p* < 0.05).

## 3. Results

### 3.1. Expression of PEDV Full Length S and S1 Protein by Recombinant Baculoviruses, S-Bac and S1-Bac

**Figure 2:**
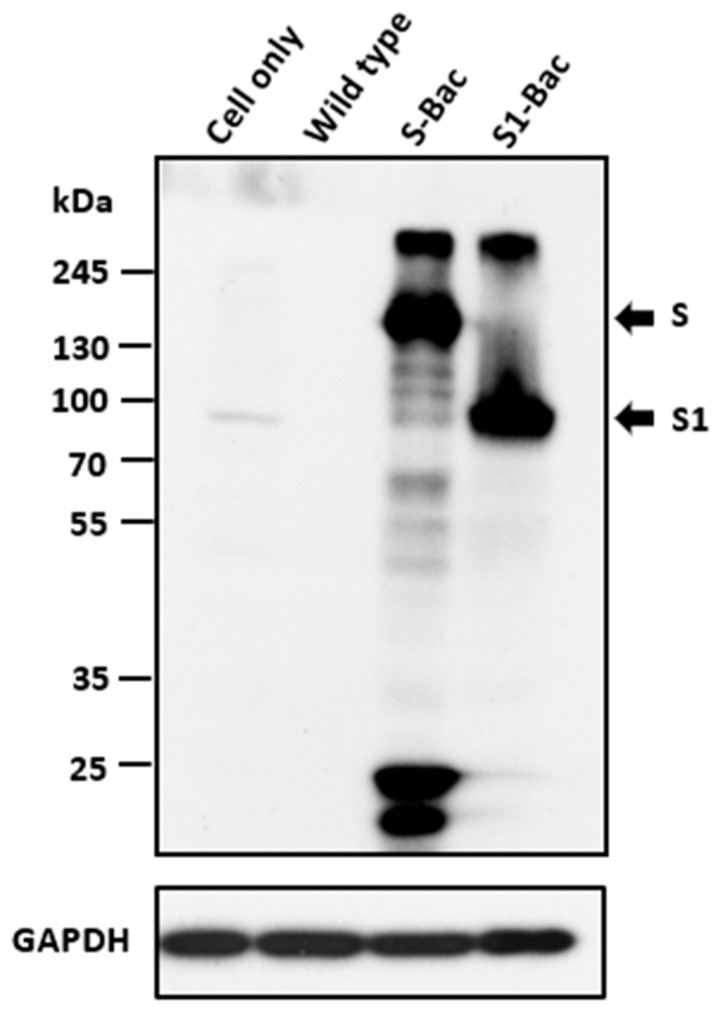
The detection of porcine epidemic diarrhea virus (PEDV) full-length S and S1 proteins in the cell lysate of S-Bac and S1-Bac infected Sf21 cells at 3 days post-infection with an M.O.I. of 5. Western blotting analysis of PEDV S and S1 proteins expressing by baculoviruses was performed and probed with anti-His tag antibodies. The corresponding molecular weights of S and S1 proteins were approximately 170–200 kDa and 90–100 kDa, respectively. Cell only: the non-infected Sf21 cell; Wild-type: Sf21 cells infected with wild type AcMNPV; S-Bac: Sf21 cells infected with S-Bac; S1-Bac: Sf21 cells infected with S1-Bac; GAPDH: control cellular protein for equal volume loading.

After propagating S1-Bac and S-Bac derived from co-transfection of pTriEx-S or pTriEx-S1 with AcMNPV baculovirus genome in the Sf21 cells, the Sf21 cells were lysed and analyzed by western blotting to evaluate the expressions of S and S1 proteins. The positive signals of the S and S1 proteins were observed at the sizes around 170–200 kDa and 90–100 kDa, respectively (Figure 2). As a negative control, no detectable signal was observed in the lysate of Sf21 cells infected with wild-type AcMNPV virus.

### 3.2. The Visualization of S and S1 Proteins Displayed on the Surface of S-Bac and S1-Bac by Electron Microscopy (EM)

**Figure 3:**
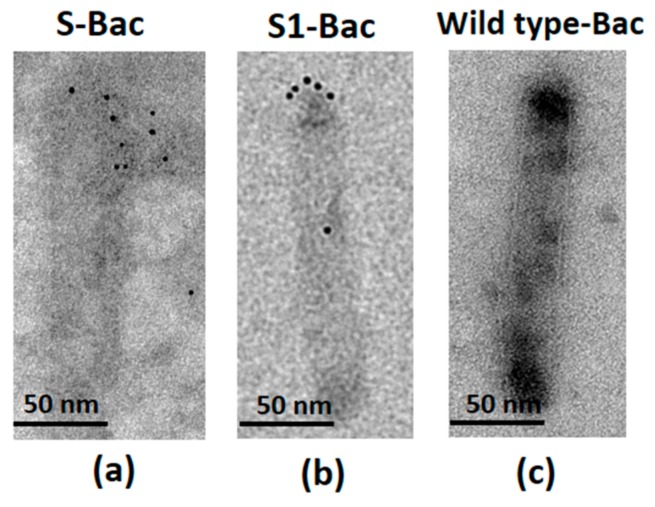
The detection of recombinant SBac and S1-Bac by electron microscopy (immuno-EM). The electron micrographs demonstrated positive colloid gold signals of porcine epidemic diarrhea virus (PEDV) full-length S and S1 proteins on the surface of recombinant S-Bac (**a**); S1-Bac (**b**); and wild-type Bac (**c**), respectively. S-Bac: PEDV S display baculovirus viral particle. S1-Bac: PEDV S1 display baculovirus viral particle. Wild type-Bac: wild-type baculovirus viral particle. The bars represent a reference of 50 nm.

To investigate whether the S or S1 proteins were displayed on the recombinant baculoviruses, the viral particles of S-Bac and S1-Bac collected and purified from culture supernatants were probed with colloid gold-labeled antibodies and examined by EM. As shown in Figure 3, the EM images revealed regular long rod-shaped virions with approximate sizes of 200 nm with clear colloid gold particles on the apex of both S-Bac (Figure 3a), S1-Bac (Figure 3b) and wild-type baculovirus (wild type-Bac, Figure 3c) virions.

### 3.3. Systemic PEDV S-Specific IgG in Mice

**Figure 4:**
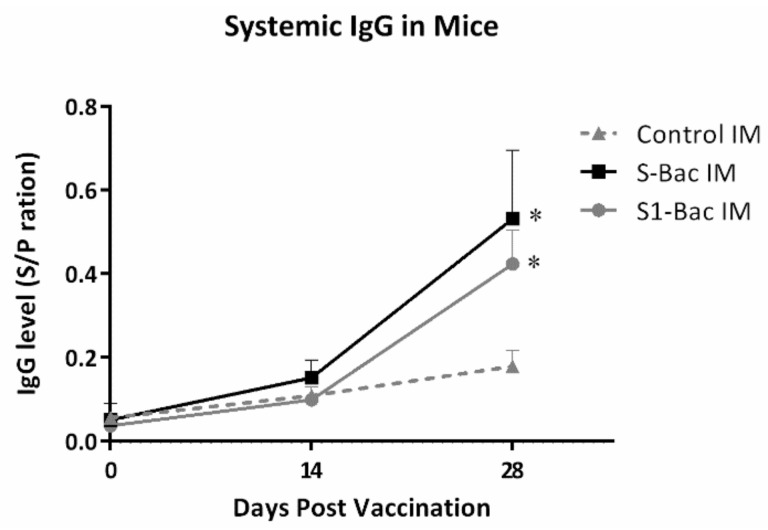
The changes of systemic porcine epidemic diarrhea virus (PEDV) spike (S)-specific IgG levels in S-Bac and S1-Bac vaccinated mice. The serum samples of the mice were collected three times in two-week intervals, including day 0 (pre-priming), 14 (2 weeks post-priming), and 28 (2 weeks post-boosting). The systemic anti-PEDV S protein IgG levels were detected by the PEDV S protein-based ELISA. The *X* axis represents the day post vaccination; whereas the *Y* axis shows the sample-to-positive control ratios (S/P ratio) of the optical density (OD) values from ELISA. The S/P ratio was defined as the ratio of the difference between the OD values of sample and negative control and the difference between OD values of positive and negative controls. The error bars represent the SD values of each group in different time points. The solid line with square icon and the gray line with round icon represent the climbing trend of IgG level in the S-Bac group and S1-Bac group, respectively. The IgG levels in the control group was expressed as the dotted line with triangle icon. *: significant difference with the control group (*p* < 0.05).

To evaluate the immunogenicity of S1-Bac and S-Bac, the PEDV S-specific blood IgG levels were determined at day 0 (pre-priming), 14 (2 weeks post-priming), and 28 (2 weeks post-boosting) in mice using a PEDV S-based indirect ELISA. The mean sample-to-positive control ratios (S/P ratio) were analyzed and are shown in Figure 4. At day 14 (2 weeks post-priming), the mean S/P ratios of systemic IgG levels in mice were 0.15 ± 0.04 and 0.1 ± 0.03 in S-Bac and S1-Bac groups, respectively, and had no significant difference from that of the control group. At day 28 (2 weeks post-boosting), the mean S/P ratios of PEDV S specific IgG levels were elevated to 0.53 ± 0.16 and 0.42 ± 0.08 in S1-Bac and S-Bac groups, respectively, and were significantly higher than that of the control group, 0.18 ± 0.04. No statistical difference of the systemic PEDV specific IgG levels was observed between the S1-Bac and S-Bac groups during the study.

### 3.4. Neutralizing Antibody Titer in Blood of Mice

**Figure 5:**
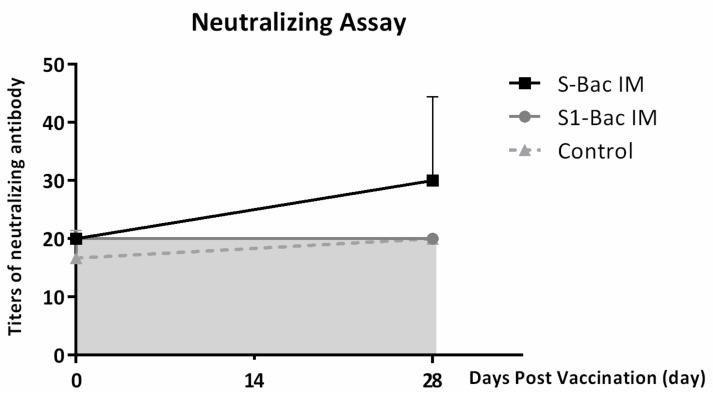
The neutralizing titers of systemic antibody of mice in control, S-Bac and S1-Bac groups at day 0 (pre-priming) and day 28 (2 weeks post-boosting). The shift of neutralizing titers of S-Bac and S1-Bac vaccinated mice are represented as a solid line with square icons and a gray line with round icons, respectively. The neutralizing titers in the blood of control mice are labeled with triangle icons on a dotted line. The error bars represent the SD values of each group at different time points. The background of this neutralizing assay was 1:20, and the area under detection background is marked as a gray zone.

The neutralizing antibodies against PEDV-PT in serum of mice were analyzed. As shown in Figure 5, there was no detectable neutralizing antibody in any of the groups at day 0 (pre-priming). At 2 weeks post-boosting, while no neutralizing antibody was detected in either S1-Bac or control groups, the average neutralizing antibody titer in the S-Bac group was elevated and reached to 1:30 ± 14.

### 3.5. Systemic PEDV S-Specific IgG and Fecal PEDV S-Specific IgA in Pigs

**Figure 6:**
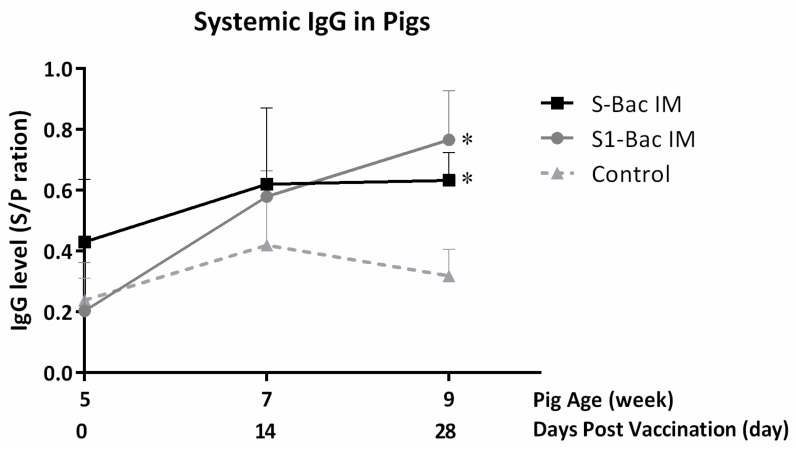
The systemic anti-porcine epidemic diarrhea (PEDV) spike (S)-specific IgG levels in piglets after S-Bac or S1-Bac vaccination. The systemic IgG levels of piglets were detected every two weeks at day 0 (pre-priming), day 14 (2 weeks post-priming) and day 28 (2 weeks post-boosting) by using the PEDV S-based ELISA. The data is shown as the mean values of the sample-to-positive control ratios (S/P ratio), which was defined as the difference between the optical density (OD) values of the sample and the negative control and divided by the difference between OD values of the positive and negative control. The error bars represented the SD values of each group in different time points. The solid line with square icon and the gray line with round icon represent the climbing trend of IgG level in the S-Bac group and S1-Bac group, respectively. The IgG levels in the control group are expressed with the dotted line with triangle icon. *: significant difference with the control group (*p* < 0.05).

**Figure 7:**
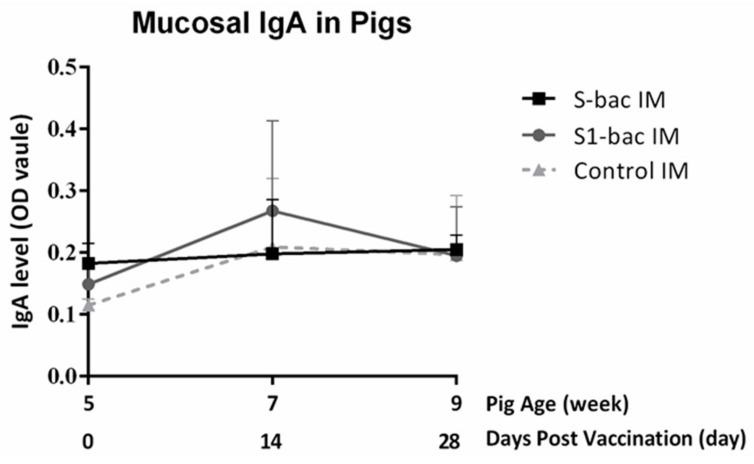
The anti-porcine epidemic diarrhea (PEDV) spike (S)-specific fecal IgA levels in piglets after S-Bac or S1-Bac immunizations. The mucosal IgA levels of pigs were evaluated every two weeks at day 0 (pre-priming), day 14 (2 weeks post-priming) and day 28 (2 weeks post-boosting) from rectal swabs by using PEDV S-based ELISA. The data is presented as mean OD values from five pigs. The error bars represent the SD values of each group at different time points. The solid line with square icons and the gray line with round icons represent the climbing trend of IgA level in the S-Bac group and S1-Bac group, respectively. The IgA levels in the control group is expressed with the dotted line with triangle icon.

To estimate the systemic immune responses against PEDVs, the plasma IgG was evaluated at day 0 (pre-priming), 14 (2 weeks post-priming), and 28 (2 weeks post-boosting). The mean S/P ratio was analyzed and is shown in Figure 6. At day 14 (2 weeks post-priming), the mean S/P ratios of systemic IgG levels of pigs in the S-Bac and S1-Bac groups secularly and slowly rise to 0.62 ± 0.25 and 0.58 ± 0.08, respectively. However, the IgG levels showed no significant differences from that of the control group. At day 28 (2 weeks post-boosting), the mean S/P ratios of IgG levels of pigs were significantly elevated to 0.63 ± 0.09 and 0.77 ± 0.16 in both S-Bac and S1-Bac group, respectively, with significant differences (*p* < 0.05) from that of the control group. As to the PEDV S-specific mucosal IgA level (Figure 7), although there was no statistically significance of fecal IgA levels in piglets among all groups during the study, the IgA level was slightly elevated in the S1-Bac group at day 14 (2 weeks post-priming).

### 3.6. Neutralizing Antibody Titer in Blood of Pigs

**Figure 8:**
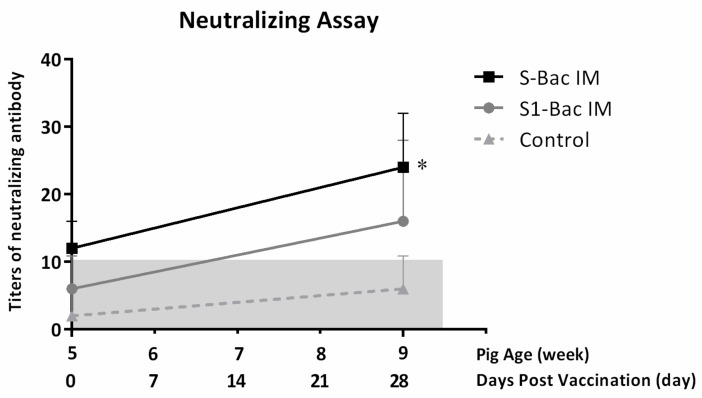
The neutralizing titers against porcine epidemic diarrhea virus (PEDV) in each group at day 0 (pre-priming) and day 28 (2 weeks post-boosting). A solid line with square icons, a gray line with round icons, and a dotted line with triangle icons represent the titers of anti-PEDV neutralizing antibodies of pigs in the S-Bac, S1-Bac, and control groups, respectively. Values are presented as means ± SD. The gray zone represents the background of the neutralizing assay. *: significant difference with the control group (*p* < 0.05).

The mean titers of neutralizing antibody against PEDV-PT strain in different groups are presented in Figure 8. During the study, different levels of neutralizing antibody against the PEDV-PT strain were detected in both S1-Bac and S-Bac immunized groups. No neutralizing antibody against the PEDV-PT strain was detected in the control group. At day 28 (2 weeks post-boosting), the neutralizing titers of the S1-Bac and S-Bac groups, 1:16 ± 12 and 1:24 ± 8, respectively, were higher than that of the control group, 1:6 ± 4.9. Interestingly, the neutralizing titers of pigs in the S-Bac group were significantly higher than those in the S1-Bac and the control groups. No significant difference of neutralizing antibody against the PEDV-PT strain was noted between the S1-Bac and the control groups.

### 3.7. Body Weights of the Pigs

**Figure 9:**
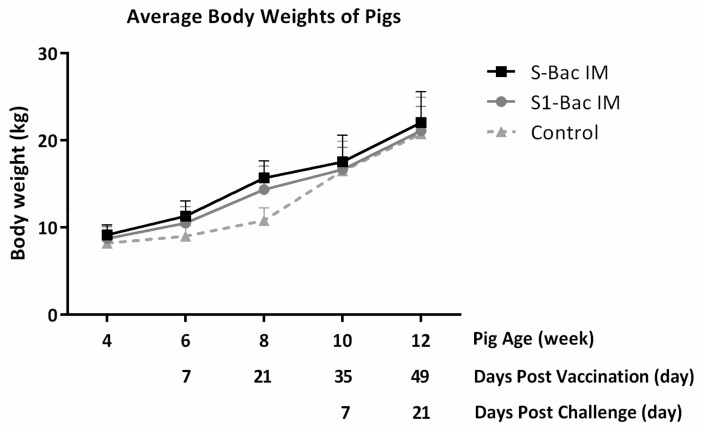
The average body weight of piglets in each group. The body weight of the piglets was measured in two-week intervals since the piglets were 4 weeks old. The *X* axis is the time line, indicating the age of the piglets, days post vaccination, and days post challenge. The piglets were vaccinated twice at 5 weeks old and 7 weeks old (labeled with white arrows); the piglets were challenged with 5 × 10^5^ TCID50 PEDVPT-P6&7 at 9 weeks old (labeled with a black solid arrow). The *Y* axis is the averaged body weights of five piglets in each group. The error bars represent the SD values of each group in different time points. The solid line with square icons and the gray line with round icons represent the average body weights of pigs in the S-Bac group and S1-Bac group, respectively. The average body weight of pigs in the control group is expressed as the dotted line with triangle icon.

During the study, the body weight of each piglet was monitored every two weeks after being introduced into the animal facility (Figure 9). Although pigs in the control group showed slightly less weight gain during the vaccination period as compared with S1-Bac and S-Bac groups, no significant difference of body weight was observed among any groups during the study.

### 3.8. Stool Scoring

**Figure 10:**
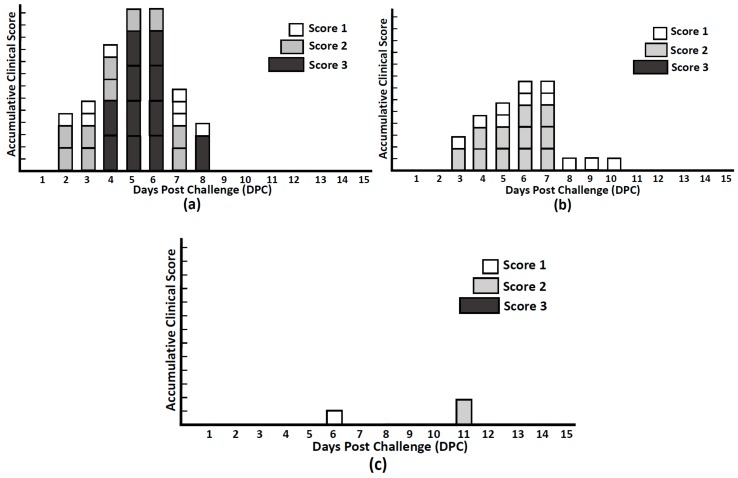
The accumulations of stool scores in the control group (**a**); S1-Bac group (**b**); and S-Bac (**c**) groups after the highly virulent porcine epidemic diarrhea virus (PEDV-PT) challenge. The clinical signs were scored by the following rules: 0, normal stool; 1, loose consistency of the stool; 2, semi-fluid consistency of the stool; 3, watery diarrhea. Each piglet was orally challenged with 5 × 10^5^ TCID50 PEDV-PT-P6&7 at day post vaccination 28 (day post challenge 0). A total of 15 days’ observation period was taken after challenge. The blank square represents the score 1 (loose consistent stool); the light-gray square represents the score 2 (semi-fluid stool); the dark-gray square represents the score 3 (watery diarrhea).

Before the PEDV-PT challenge, no clinical signs were observed in any groups. After orally challenging pigs with PEDV-PT-P6&7 (Figure 10), three of five pigs (3/5) in the control group presented mild to moderate diarrhea, which was scored as 1 to 2, at 2 days post challenge (DPC). At 4–7 DPC, all pigs in the control group showed moderate to severe clinical signs. During the study, all pigs in the control group had 5–6 days of watery diarrhea (score 3) and recovered at 9 DPC. Comparatively, the S-Bac and S1-Bac immunized pigs showed a decrease in the overall severity of diarrhea, a delayed onset of the disease, and a shortened course of illness as compared with pigs in the control group. In the S1-Bac immunized group, the appearance of clinical signs was postponed to 3 to 4 DPC, and all pigs showed milder symptoms, which were scored at 1 to 2, as compared with the control group during the study. In the S-Bac immunized group, importantly, four of five (4/5) pigs presented no clinical symptoms during the study, excepting one pig had soft feces (score 1) at 6 DPC and semifluid feces (score 2) at 11 DPC.

### 3.9. Fecal Viral Shedding

**Figure 11:**
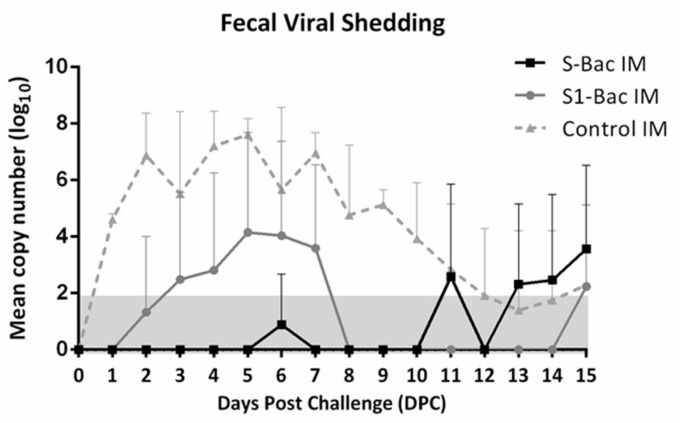
The detection of fecal viral shedding after the highly virulent porcine epidemic diarrhea virus (PEDV-PT) challenge. The limit of detection for this probe-based quantitative real-time RT PCR was 1.8 log^10^ and is labeled as the gray zone. The error bars represent the SD values of each group at different time points. The solid line with the square icons and the gray line with round icons represent the average fecal viral shedding copy number of pigs in the S-Bac group and S1-Bac groups, respectively. The average fecal viral shedding copy number of pigs in the control group is expressed as the dotted line with the triangle icons.

The viral shedding of PEDV detected by a PEDV N-based real-time RT PCR is presented in Figure 11. The pigs in the control group started to shed PEDVs into the stool with the mean value of the copy number of 4.6 ± 0.19 log_10_ copies/mL at 1 DPC, continuously increasing and fluctuating over 3 to 8 DPC, with peak viral shedding of 7.6 ± 0.57 log_10_ copies/mL at 5 DPC, and declining after 8 DPC. After 12 DPC, the amount of viral shedding in most of pigs in the control group was below the detection limit. In the S1-Bac immunized group, the virus started to be detected with the mean copy number of 2.5 ± 3 log_10_ copies/mL at 3 DPC, lasted for 5 days, with peak viral shedding of 4.2 ± 3.5 log_10_ copies/mL at 5 DPC. Importantly, most pigs in the S-Bac group had no detectable viral shedding during 0–10 DPC, excepting 1 pig, who exhibited an intermittent viral shedding of 4.5 log_10_ copies/mL at 6 DPC and 7.7 log_10_ copies/mL at 11 DPC. Comparing with the control group, the amount of fecal viral shedding in the S-Bac group was statistically significantly lowered (*p* < 0.05).

## 4. Discussion

Two novel recombinant baculoviruses displaying the full-length S protein (S-Bac) and the S1 domain (S1-Bac) of PEDV-PT were successfully generated in this study. While both S1-Bac and S-Bac are capable of eliciting systemic PEDV S-specific IgG in mice and piglets, significant induction of neutralizing antibodies against PEDV-PT strain could only be detected in animals immunized with S-Bac. After orally challenging with PEDV-PT-P6&7, the S-Bac and S1-Bac vaccinated piglets present milder clinical symptoms with significantly lower fecal viral shedding than that of piglets in the control group. Importantly, intramuscular vaccination with S-Bac elicits superior protection against PEDV over intramuscular S1-Bac vaccination in piglets. Circulating in North America and Asia, the G2b PEDV strains exhibiting higher pathogenicity than that of G1 PEDV strains are responsible for recent outbreaks [42,43,44]. The S-Bac vaccine based on based on the full-length of S protein of the G2b Taiwan PEDV-PT strain might be a good candidate for controlling the outbreaks of highly virulent G2b PEDVs.

Utilizing baculovirus display system to express the antigens as vaccine candidates is one of the brilliant applications of baculovirus. This approach is generally achieved by cooperating the protein of interest with the baculoviral envelope glycoprotein, GP64, for anchoring the protein on the membrane of the budded virions [45]. Different surface displaying strategies, such as changing signal peptide, transmembrane domain (TM) or cytoplasmic tail domain (CTD) of GP64 in attempt to present large, complex foreign proteins as well as simple peptides are available [29,46]. In the present study, using the traditional chimeric GP64 display strategy, which consists of PEDV S1 domain fused with the TM and CTD of GP64, the S1-Bac has been successfully generated. Importantly, the S-Bac displaying the full-length of PEDV S protein without being fused to GP64 TM and CTD has also been successfully generated and confirmed. To our knowledge, this is the first evidence showing the full-length S protein of coronavirus could be successfully displayed on the surface of the baculovirus by utilizing PEDV-S original TM and CTD. Our study provides the rational basis of using baculovirus to display the full-length S protein of coronavirus. This approach is a novel tool for developing vaccines against other coronaviruses for better antigen delivery, such as severe acute respiratory syndrome coronavirus (SARS-CoV) and Middle East respiratory syndrome coronavirus (MERS-CoV).

Currently, two conditionally licensed PED vaccines, the alphavirus-based vaccine (Harrisvacccines, Inc., Ames, Iowa, USA) and an inactivated vaccine (Zoetis, Inc., Parsippany ,New Jersey, USA) targeting the highly virulent G2b PEDV are available in the United States. However, they are not available in other countries. The former vaccine is a PEDV RNA vaccine, and previous results showed that application of the vaccine could only reduce mortality of piglets by 3% [8]. The second one used killed PEDV viruses, and a low viral yield with a titer of 6.6 log10 TCID50/mL is limiting the broad application of the vaccine [8]. Recently, several studies using full-length ectodomain trimeric S protein [40] or different truncated S proteins derived from different G2b PEDVs were combined with commercial adjuvants as subunit vaccine regimens. Despite that these subunit vaccines were able to induce robust systemic IgG and neutralizing antibody against PEDVs, they fail to completely prevent clinical symptoms and fecal viral shedding [47,48,49]. In the present study, piglets vaccinated with S-Bac can be elicited higher neutralizing antibody titers and good protective efficacy over the S1-Bac vaccinated pigs, despite both of them promoting robust levels of systemic PEDV S-specific IgG in pigs and mice. The remarkable differences of protective efficacy between S1-Bac and S-Bac vaccinations suggest the importance of the integrity of the S protein in inducing neutralizing antibodies and protective immunity. The S protein of PEDV is the most important viral protein for establishing infection and eliciting neutralizing antibodies in hosts. The S1 domain is majorly responsible for virus-host recognition and interaction, and the S2 participates in membrane fusion and virus internalization [17,50]. Several PEDV neutralizing epitopes in S1 domain have been reported, such as the domain 0, domain B [14], and the CO-26K equivalent epitopes (COE) [15]. On the other hand, the S1D epitope, covering the C-terminus of the S1 region, the S1/S2 junction, and the N-terminus of the S2 region, is also verified as an effective neutralizing epitope of PEDV [19]. However, there are also some other neutralizing epitopes of PEDV identified in S2 and the CTD of S protein, such as the 2C10 epitope at the C-terminal site [18] and several linear epitopes at the N-terminal site of the S2 region near the SS2 and SS6 epitopes [51]. Our results of superior immune-protectivity of the S-Bac over the S1-Bac vaccination indicate that the baculovirus display S protein comprising both S1 and S2 regions is crucial for generating protective immunity against PEDVs.

It has been well-demonstrated that baculovirus itself can serve as a strong B and T cell adjuvant for protein antigens [27,52]. Further, the viral particle of baculovirus is able to evoke robust innate immune responses by regulating cytokines such as interleukin 6 (IL-6) and tumor necrosis factor alpha (TNF-α) [26], which may take place in blocking the viral infection. In our study, we demonstrated that both S-Bac and S1-Bac immunizations without any adjuvant could evoke PEDV S-specific systemic immune response, ease the clinical symptoms, and decrease the viral shedding to a certain extent. Although S-Bac immunization elicits robust systemic immunity and almost completely protects piglets against PEDV-PT-P6&7 challenge, there is still one in five piglets exhibiting mild symptoms and delayed low level of viral shedding. Concerning the detectable but low level of fecal IgA in both S-Bac and S1-Bac immunized piglets, new regimens to induce mucosal immunity are critical for PEDV vaccine development [53,54]. For instance, introducing the specific adjuvants, such as LTB and CTB (cholera toxin B), along with baculovirus displayed antigens to propel the mucosal and cellular immunity [34,55]. For further experimental design of an intramuscularly injectable vaccine, the utilization of adjuvants which are capable of either boosting immunity or elevating common mucosal immunity should be considered.
